# Supplementary material for: Development and evaluation of a java-based deep neural network method for drug response predictions
Source: Front Artif Intell. 2023 Mar 23;6:1069353. doi: 10.3389/frai.2023.1069353 (PMC10076891; doi:10.3389/frai.2023.1069353)
Supplement: Supplementary file 1 [file Data_Sheet_1.PDF]

Supporting Information

Architectures of JavaDL in XML

```
<lay1 nodes_num_in="182" nodes_num_out="200" activation="tanh"
dropout_rate=0.5/>
<lay2 nodes_num_in="200" nodes_num_out="200" activation="tanh"/>
<lay3 nodes_num_in="200" nodes_num_out="200" activation="tanh"/>
<lay4 nodes_num_in="200" nodes_num_out="1" activation="identity"/>
```

Architectures of CNN

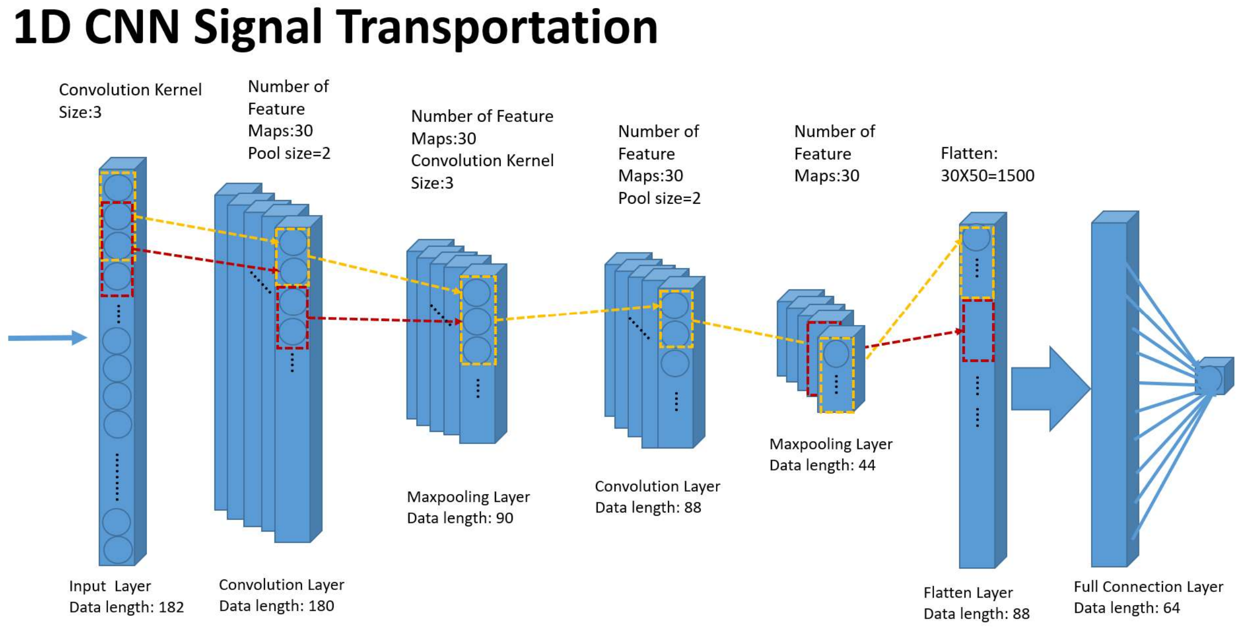

Learning rate = 0.01, Batchsize = 25, Momentum = 0.1
The cost function is described and implemented based on **Eq.3**. The optimizer for both JavaDL and CNN is Adam. The source code for training the JavaDL and CNN models are implemented with Deeplearning4j 1.0 (Java 1.8) and keras 2.0 (Python 3.6).

For the usage of the package of the prediction model: The file for input parameters for training data set HCC_1937 is named “HCC_1937.xml”. The node labeled “net” denotes the configuration of the deep neural networks. It is also available online for download, along with other example files: https://www.imdlab.net/JavaDL/.
